# Development of a Non-Contact Sensor System for Converting 2D Images into 3D Body Data: A Deep Learning Approach to Monitor Obesity and Body Shape in Individuals in Their 20s and 30s

**DOI:** 10.3390/s24010270

**Published:** 2024-01-02

**Authors:** Ji-Yong Lee, Kihyeon Kwon, Changgyun Kim, Sekyoung Youm

**Affiliations:** 1 Center for Sports and Performance Analysis, Korea National Sport University, Songpa-gu, Seoul 05541, Republic of Korea; 302479@knsu.ac.kr; 2 Department of Information & Communication Engineering, Kangwon National University, Samcheok 25913, Gangwon-do, Republic of Korea; kweon@kangwon.ac.kr; 3 Department of Artificial Intelligence & Software, Kangwon National University, Samcheok 25913, Gangwon-do, Republic of Korea; 4 Department of Industrial and Systems Engineering, Dongguk University, Seoul 04620, Republic of Korea

**Keywords:** body generation confidence, human shape estimation, synthetic dataset, generative adversarial network, obesity

## Abstract

This study demonstrates how to generate a three-dimensional (3D) body model through a small number of images and derive body values similar to the actual values using generated 3D body data. In this study, a 3D body model that can be used for body type diagnosis was developed using two full-body pictures of the front and side taken with a mobile phone. For data training, 400 3D body datasets (male: 200, female: 200) provided by Size Korea were used, and four models, i.e., 3D recurrent reconstruction neural network, point cloud generative adversarial network, skinned multi-person linear model, and pixel-aligned impact function for high-resolution 3D human digitization, were used. The models proposed in this study were analyzed and compared. A total of 10 men and women were analyzed, and their corresponding 3D models were verified by comparing 3D body data derived from 2D image inputs with those obtained using a body scanner. The model was verified through the difference between 3D data derived from the 2D image and those derived using an actual body scanner. Unlike the 3D generation models that could not be used to derive the body values in this study, the proposed model was successfully used to derive various body values, indicating that this model can be implemented to identify various body types and monitor obesity in the future.

## 1. Introduction

In healthcare, various body values are used to evaluate information such as body type, obesity, and body balance. Parameters such as chest volume and waist volume are used to derive body shape information through waist circumference, chest circumference, and thigh circumference or to determine obesity [1]. Thus, body volume, circumference, and cross-sectional area representing various body characteristics are increasingly used to derive body information that cannot be measured using the existing obesity and body shape indices [2]. In addition, the accessibility of a vast amount of previously unavailable medical data has expanded the information provision of digital healthcare [3]. In the past, body value derivation was measured by measuring body information using a tape measure using a measuring expert and measuring length through a two-dimensional (2D) image. These traditional methods of body measurement, such as using scales, body composition analyzers, and calorie calculators on smartwatches, can sometimes yield errors influenced by the measurement environment and the individual conducting the measurements [4]. To enhance accuracy and reduce these errors, various advanced sensors have been developed. Among these, 3D body scanners stand out as a significant innovation. They are capable of measuring body dimensions, including length, girth, volume, and cross-sectional area, without the need for professional assistance. Three-dimensional (3D) data obtained from these scanners offer a detailed insight into an individual’s body type, level of obesity, and overall body balance, providing a more comprehensive and accurate health assessment.

Accurate body measurement and assessment without the need for professional assistance require detailed body information, making 3D body data increasingly essential in the field of digital healthcare [5]. However, acquiring 3D data often necessitates expensive equipment, which can be bulky and heavy, limiting the accessibility of low-cost digital healthcare solutions. To mitigate these drawbacks, a method involving the use of 3D kinematic cameras and multi-image composition has been developed. This approach, however, involves capturing objects beyond the body, necessitating post-processing techniques such as background removal, hole filling, and 3D smoothing [6].

Additionally, 3D transformation algorithms have been extensively researched. In particular, Amazon’s Halo leverages technology that converts 2D images into 3D body data to comprehend users’ body shapes and assess crucial health indicators such as obesity and body fat percentage. This technology has been commercialized in recent years, with many individuals experiencing the precise transformation of 2D images into accurate 3D body data through the Halo service. Building upon these experiences, the technology of converting 2D images into 3D data underscores innovative advancements in the fields of health management and body shape assessment. These algorithms facilitate the creation of 3D objects by synthesizing multiple images and identifying the coordinates of 2D body joint positions. This process effectively overcomes the limitations of multi-dimensional 2D constraints, optimizing 3D creation. Such advancements in 3D technology not only enhance the precision of digital healthcare but also broaden its potential applications [7].

These diverse methods for body measurement and analysis are often complex and come with significant limitations for general use. In healthcare, particularly for obesity and body shape management, there is a need for regular monitoring and management of one’s body. However, various sensor equipment capable of measuring body metrics tend to be quite expensive and are typically only available at specialized hospitals or centers. Furthermore, even when 3D data generation is possible, there are considerable challenges in processing these data to derive body values accurately, presenting additional barriers to widespread and practical use in healthcare contexts [8]. However, while there are many studies analyzing predictions of diseases or detecting outliers based on body values, there is a lack of research that can derive diverse and accurate body values from a limited amount of body information. Recognizing accurate body information is of paramount importance from a healthcare perspective and can serve as a valuable tool for the continuous management of conditions such as obesity and body shape.

Therefore, if you can simply derive complex body information such as 3D body data, you can visually understand your various body information properly, obtain body type and body balance information, and numerically quickly identify imbalance and hypertrophy of specific body parts [9]. More accurate healthcare can be provided by quickly determining incorrect posture and insufficient body parts.

In this study, we introduce a method to create 3D body models using just two images—front and side views—taken with a standard mobile phone camera. This method is part of our system designed to generate 3D bodies and derive various body values, which can be used regardless of time and place. This approach allows for easy, anytime-anywhere self-filming without the need for expensive or complex contact equipment. Propose a non-contact sensor system that generates 3D data from these 2D images, extracts various metrics from these 3D body data, and applies this technology in healthcare for continuous body management and monitoring. The aim is to facilitate body management through regular, convenient, and non-invasive assessments.

## 2. Methodology for Deriving Detailed Body Information

### 2.1. 3D Generation Using Non-Contact Sensors and Equipment

To date, several studies have been conducted on the creation of 3D objects using accurate 3D models and methodologies that reduce the complexity of the 3D creation process. In 2000, Cheung et al. [10] extracted 2D images using multiple PCs and cameras installed in 360° zones and constructed a body model using image data of more than 15 frames per second; a maximum of 299 frames were used for the frame, and a 3D model was created for movements. In the same year, Paul et al. [11] created a 3D human body model with a realistic appearance using a non-contact optical sensing technique called C3D. However, the two technologies described above require a large space and considerable time for shooting.

There are many restrictions on accurate 3D generation, and many complexities appear. Therefore, various studies have been performed by different authors to solve these issues. In 2002, Belongie et al. [12] gathered and analyzed synthetic data generated by modeling and simulation and those collected in a laboratory environment using three methods, viz. the discriminative method, general method, and photographic structure method. Additionally, 1100 behavioral data points were used to construct a human body model. In the discriminative method, silhouette contours are generated in each frame using discrete cosine transform (DCT) [12] and replacing shape context [13]. Simultaneously, a contour is created with DCT to perform body modeling for walking and crawling behavior. In 2015, Cheng et al. [14] evaluated the contours of front and side images using the generative method and combined the pixel values of the contours into pixels in the model contour to create a single-body model. In the photographic structure method, data for body parts were extracted from the histogram using the oriented gradient method and expressed by dividing them into features for each part. In 2018, Su et al. [15] created a new 3D model by restoring a previous 3D model, using the SPLATNet model for buildings created with 2D and 3D images in the form of a point cloud. The object was divided, and each part was recognized and implemented as a new 3D model containing a 2D–3D layer [15]. Further, in 2018, Pavlakos et al. [16] conducted a study to judge human behavioral poses and models using a 3D human pose model. The joint movements were measured, and a model that predicted the human behavior pose was created. For predicting the poses, MoCap data for the pose source and body scan data for the shape source were used, along with various behavioral datasets [16].

### 2.2. 3D Generation Using Deep-Learning Models

Machine learning is a way to solve new problems through datasets. Recently, numerous studies related to data generation and the creation of 3D objects with multiple 2D images have been conducted. To further improve the performance of 3D generation, Xiang et al. [17] evaluated landmarks for 3D objects through PASCAL3D+ datasets, thereby predicting the depths in 2D images and creating similar 3D models. Therefore, by creating a model using PASCAL3D+, the landmarks for each object were presented, and based on this, a 3D object was created. In 2019, Zheng et al. derived the feature part of data using evaluated 3D body object images as input and created a virtual 3D model based on the derived silhouette [18].

In addition, based on the productive adversarial neural network model, a method of generating textures for each body part and combining them with a virtual model to generate one 3D body model was presented. In 2020, Van et al. used a 3D camera and created a neat 3D model through rendering after preprocessing the derived body model [19]. In 2021, Dwivedi et al. identified the features in a single image based on model-provided skin information (skinned multi-person linear model (SMPL)) for similar 3D body data and then created a virtual 3D model similar to the image using adjacent meshes [20].

### 2.3. Methodology for Deriving Detailed Body Measurements Information

In the past, mainly traditional methods were common, i.e., direct measurements of body parts using a tape measure. In parallel, research was conducted to determine body proportions and shape through 2D image analysis [21]. This method was prone to errors depending on the user’s measurement technique and posture, and 2D images had limitations in fully understanding the 3D structure of the body. Although various studies have been conducted on methods to measure body information, there were limitations in measuring detailed information in the past. For this reason, in 2011, body mass index (BMI) information was derived using only height and weight, which can be most easily measured, and various studies were conducted using that information [22]. In 2012, research was conducted to detect each part of a person using three Microsoft Kinect cameras capable of capturing 3D information and synthesizing them to create 3D data. In this study, body information was extracted using a passive method of synthesizing captured 3D data and reconstructing empty space [23]. Since 2019, research on the development of measurement and sensor systems using modern mobile phones has been published. The use of smartphones as sensing systems continues to attract interest due to their powerful computing power, rich set of embedded sensors and integrated wired and wireless communication technologies. Therefore, the widespread adoption of smartphones, even in developing countries, provides an opportunity for the development of low-cost sensor systems, especially in healthcare [24]. In 2020, BMI was measured in more detail based on exercise ability and a person’s physical performance. In measuring this detailed BMI, various behaviors were filmed with a camera, collected, and used for analysis [25]. In 2022, using smartphone cameras and wearable inertial measurement units (IMUs), we estimated the knee adduction moment (KAM) and knee flexion moment (KFM) to develop a model to optimally diagnose walking to reduce knee load due to walking [26].

This recent trend is to measure and analyze body information using portable devices such as smartphones, including 3D modeling using high-resolution cameras, health monitoring using built-in sensors, and extraction of body measurements using image processing and machine learning algorithms. Implemented in a variety of ways, it provides a low-cost and user-friendly healthcare solution. However, this is difficult for users to use, and it takes a lot of time and money to handle these data. Additionally, to collect 3D body data, a contact sensor is required, which causes a lot of discomfort to users.

Therefore, in this study, using a smartphone camera, users can simply take pictures without being limited by time and cost to generate 3D body data for visual self-diagnosis and extract various body values automatically extracted from 3D body data. Therefore, we are developing a non-contact body information measurement system that can perform self-diagnosis using 3D data and various body measurements.

## 3. Training Data

### 3.1. Data Selection

This study generates 3D body data using 2D body images and derives various body information based on these generated 3D body data. Therefore, a 3D generation model that can reconstruct a 2D image is used. To reconstruct a 2D image into 3D, 2D body image data and 3D body data must be collected as a pair. In this model design, training data are required to create a 3D body using 2D information. Therefore, 3D body data forming a pair of bodies that appear in the 2D image as well as the 3D image were selected. Since data regarding the training model should have the characteristics of the Korean-style body, training data used full-body scanned 3D data provided by Size Korea (https://sizekorea.kr, accessed on 10 November 2023). These whole-body scanned data consisted of a total of 400 data points (200 for men and 200 for women) for subjects in their 20s and 30s. Size Korea data were approved on 25 April 2020 by submitting an agreement on the Size Korea website (https://sizekorea.kr/about/conslt/data, accessed on 10 November 2023). Size Korea data are public institution data; anyone can read them and browse these data by moving to the 3D human body shape in the Find Human Information tab.

The 3D data format provided by Size Korea was the OBJ data format, which is one of the formats of 3D data providing information on 3D geometry, color, and textures as shown in Table 1. However, the OBJ datasets contained large data sizes owing to the increased complexity due to the inclusion of many numerical values. However, in this study, only 3D geometry values were required to derive the body values; therefore, the size of these data was reduced by using the STL data format that does not contain information on colors and textures and only yields details about the 3D geometry.

The findings in Table 1 indicate that while various file formats are used in 3D modeling, depending on the scanner and software involved, the STL format stands out due to its lack of color and texture information. This attribute significantly reduces the file size compared with other common 3D file formats. Consequently, STL files, with their smaller capacity yet adequate representation of 3D information, were chosen for our study. This choice aids in effectively conveying crucial 3D body data points while simplifying the training process.

Additionally, our approach involves pairing 3D body data with corresponding 2D images. This pairing strategy facilitates the reconstruction of the input image when a 2D image is provided, thereby enhancing the training and application scope of our study.

### 3.2. Data Preprocessing

**Figure 1:**
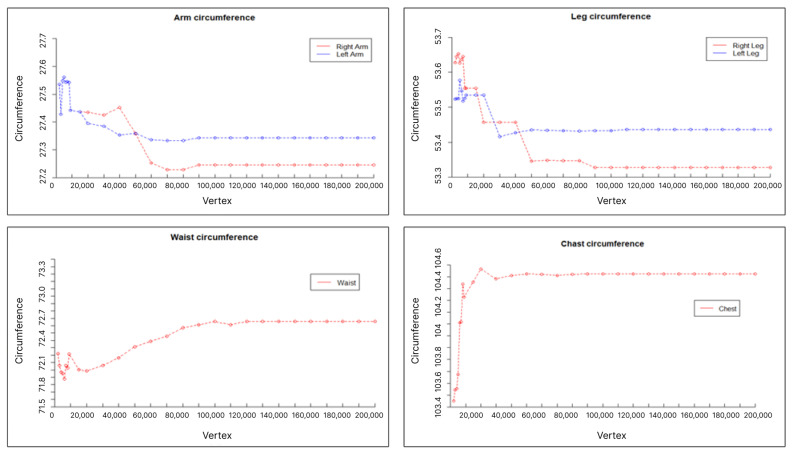
Vertex reduction: Body 3D data information loss value.

**Figure 2:**
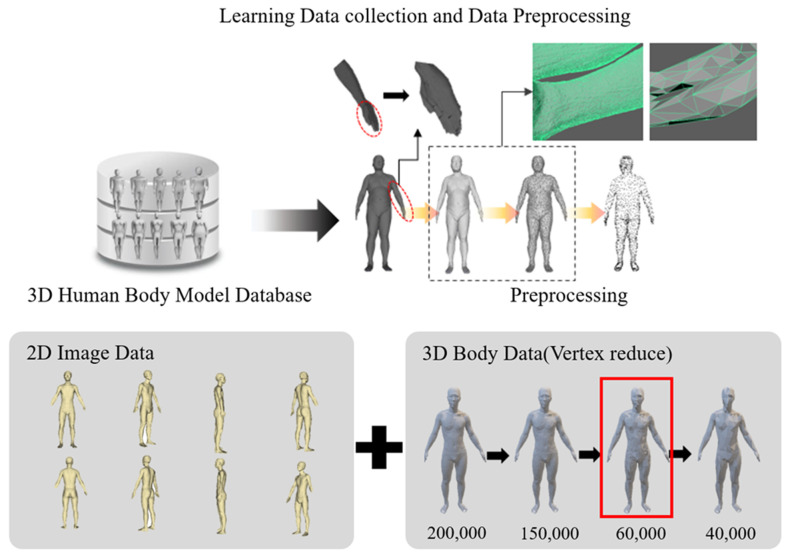
Evaluating data collection and data preprocessing process.

When training from collected data, if the volume of training data is too large, the training speed may decrease. These collected body data are 3D data consisting of dots and lines. There are 400 samples, each consisting of 200 men and 200 women, with an average of about 250,000 vertices. The STL file format was used to reduce the size from 200 MB to 15 MB, but training a total of 400 data points requires a large amount of memory and time, and it is difficult to derive an appropriate training model. Therefore, to solve the problem of massive training volume, the amount of data was reduced by reducing the number of vertices to prevent body information from being lost. Therefore, as shown in Figure 1, the size of the vertices was repeatedly reduced by 10,000, and when the number of vertices was less than 60,000 on average, body information was lost in the existing 3D data. Therefore, as shown in Figure 2, the data size was reduced from an average of 250,000 vertices to an average of 60,000 vertices and implemented as training data. Additionally, our goal was to generate 3D body data using 2D body images as input. Accordingly, a total of eight 2D image datasets around the body in 45° steps were generated for these 3D training data. In summary, one 3D body dataset and eight multi-dimensional body image datasets were paired and used to train a generative model.

Therefore, the dataset to be evaluated in this study was studied based on 3D body data. Looking at Figure 2, one piece of 3D body data was taken and rotated at a 45-degree angle, and a total of eight pictures were taken. The eight photos obtained in this way were combined with original 3D body data to form a training data set. By training images from various angles and each pair of 3D body data obtained from them, it becomes an essential basis for developing the ability to reconstruct a 3D body dataset based on images when only 2D images are input. 

## 4. Experiment and Analysis

### 4.1. 3D Generative Model Using 2D Body Images

In this study, we have two primary objectives. The first goal is to generate 3D body data using 2D body images. To achieve this, training occurred on a data set that combined 2D body image data with 3D body data. The crux of our approach lies in reconstructing these images into 3D body data when a new 2D body image is input. For this purpose, models that were most effective in efficiently generating 3D data from 2D images were selected.

A total of four models were selected—two 3D generation models using multiple images and two 3D generation models using single images—in the form of training data. Multiple images are used to generate new 3D datasets by avoiding the complexity of training 3D data values directly from 3D feature generation. By reducing unnecessary information through vertex reduction in existing 3D body data, we reduced the size of the training data and reduced the complexity of the training. In addition, we were able to improve the accuracy of the generation because we used 3D data and 2D body images that can be used in pairs for training instead of using only 3D body data. Next, an SMPL model was created, which is a 3D method, as a multi-level pixel-aligned implant function for a high-resolution 3D humanization (PI) model that can reduce the complexity and speed of 3D creation by adding volume features to 2D images.

#### 4.1.1. 3D Recurrent Reconstruction Neural Network

The 3D recurrent reconstruction neural network (3D-R2N2) is a method of generating 3D data (*n* × *n* × *n*) based on matrix training in a 3D space using a 2D input, such as RNN, implemented on a 2D dataset. The 2D image derived from various viewpoints is restored to a 3D voxel model by the input. The 3D-R2N2 model consists of an encoder receiving 2D information and a decoder deriving the 3D model restoration [27]. This model converts the 2D long short-term memory (LSTM) image into a feature vector and inputs it into a 3D long- and long-term circulating neural network model to derive the model restoration value [28]. Further, 3D-R2N2 includes a recurrent network structure, and the encoder converts the input value to a low-dimensional feature vector from the CNN. The transformed vector is transmitted to the input of the 3D convolution LSTM, so multiple inputs for the same object are accumulated. As the training progresses, a 3D object with increasing sophistication can be obtained. Therefore, this model was used to evaluate eight image datasets from various angles and 3D body datasets.

#### 4.1.2. Point Cloud GAN

In general, 3D components are composed of many points(vertex), edges, and faces, limiting their calculation and visualization due to increased complexities. To compensate for this problem, a point cloud is used to construct 3D data using a small data point (vertex) [29]. A 3D model can be created and transformed using the point cloud GAN (PointGAN) model, which learns using the 3D point values, thereby reducing the training time by reducing the traffic. Therefore, existing 3D body data was converted into a point cloud format for the general model training. In this case, N points that were not arranged in the point set of 3D body data were extracted with features in the X, Y, and Z coordinates through the feature extraction component. In addition, the up-down-up expansion unit is used to obtain the optimal object value by sampling the points that fit the characteristics.

#### 4.1.3. SMPL Model

The SMPL model is a method of finding virtual datasets using the vertices, i.e., N body meshes and K joint features. In this study, N = 6890 and K = 23 joints were used, and data used in the model consisted of 1700 men and 2100 women with shapes for various body models [27]. Therefore, it was possible to generate one virtual body data by deriving the body shape of the body image and finding the coordinate value for the joints of the derived body shape. The SMPL model registers the RGB-D sequence of a person to solve the problem of incompleteness of 3D generation through a single image and converts the shape information into a personalized shape for each sequence value of these 3D data to solve the problem of incompleteness of monocular setup. In 3D generation through 2D images, the shape and pose parameters, as well as global translation, are optimized, the shape is regularized, and 3D data similar to the finally regressed image are generated.

#### 4.1.4. Multi-Level Pixel-Aligned Implicit Function for High-Resolution 3D Human Digitization

When performing 3D body modeling, a deep training model that uses multiple cameras or calculates high dimensions to process high-dimensional data is used. However, this method provides much lower quality than that obtained with a professional capture system. In addition, since the composition and shape of the camera used to capture the 2D image must be accurately displayed in the body creation, a large amount of traffic occurs in 3D training, making it impossible to learn with high-resolution images. Therefore, training proceeds through low-resolution images, and for this reason, a proper model cannot be generated.

Therefore, the pixel-aligned impact function for the high-resolution 3D human digitization (PIFuHD) model was used to compensate for the limitations of model generation through low-resolution images [30]. The PIFuHD model consists of two processes as follows: When input data are included, an image with a 512 × 512 resolution is received as the input, and a low-resolution image of (128 × 128) is obtained. The low-resolution images are derived through multi-layer perceptron, and in other processes, input images are generated through image-to-image translation to create high-resolution front/back images. Using the generated images, high-resolution features were derived from the PIFu layer, and a 3D model was finally created through restructuring [31]. Therefore, it is possible to generate body data by reducing the complexity of the model and creating a 3D model based on the features of the 2D image through image translation without using extensive 3D training.

#### 4.1.5. Multi-High-Resolution Feature Combination Generation Network

Although the PIFuHD model can express body characteristics, since it is a 3D generation technique using a single image, it can only obtain information from the front image, leading to the loss of information on the side of the body. Therefore, compared with the conventional PIFuHD technique, the algorithm developed in this study created a more accurate model for measuring the body values by removing the background of the image and adding more side volume information. The background of the image was removed through a fully convolution segmentation of the 2D image, thereby improving the 3D generation accuracy. In addition, accurate information on the side portion of these 3D data was added by deriving and synthesizing 3D body data for the front and side values of the image.

**Figure 3:**
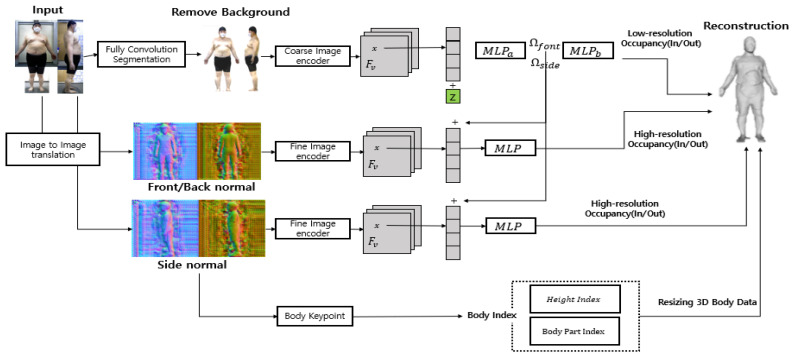
Multi-high-resolution feature combination generation network process.

Unlike the 3D generation model that uses only the front image as shown in Figure 3, the model—multi-high-resolution feature combination generation network—proposed here uses the 2D image for the front and side as the input. The human pose segmentation function uses the front and side images as the input to reduce the complexity of 3D data generation by leaving only the human silhouettes and removing them. Based on the removed background, 3D body data of the front and side are generated, and side information is synthesized using a 3D model generated as a side image for a part where side information is not expressed based on the front of the generated 3D model. Similar to the actual ratio of synthesized data, 3D data having a size similar to that of the actual value were generated through resizing.

The process of the 3D generative model proposed in this study, as shown in Figure 3, consists of an image feature extraction part and a creation part. The problem of information loss about the body image was solved by removing the background of the image, finding the feature values through embedding on the front and side and converting these values to 1 or 0 for the human shape through image translation in the 3D generation. This creates volume information using images with a convex effect on the image and creates a 3D model by synthesizing the feature parts with the front and side values. Therefore, we solved the problem of lateral data loss by remembering and synthesizing the body characteristics of the input value and using the front and side information. Furthermore, for the reconstruction model, we resized 3D body data to match the actual size using the body part index obtained from the key information and the body keypoint values derived from the body index. Finally, we converted these data into a format that can yield various body values.

### 4.2. Feature Value Extraction Model for 3D Body Data

**Figure 4:**
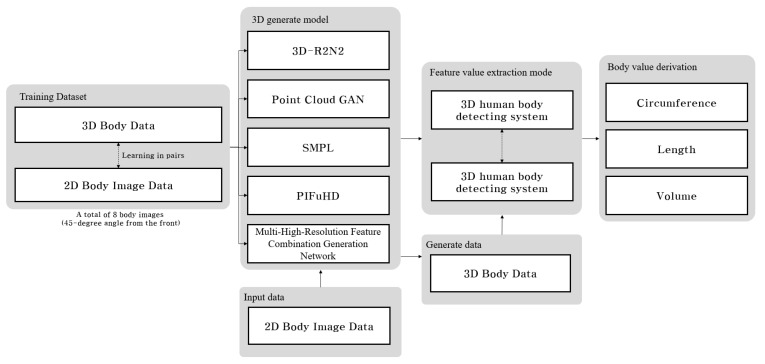
3D body creation and body data derivation process.

In this chapter, we designed a system that derives values that can be used in healthcare from 3D body data generated from the 2D body image in Section 4.1. Therefore, the body features of generated 3D body data are found, keypoints for the body features are extracted, and major body part values are derived based on the extracted keypoints. The design structure of the system is shown in Figure 4.

#### 4.2.1. Development of a 3D Human Body Detecting System

A designed system that derives values that can be used in healthcare from generated 3D body data. For system design, it is necessary to find body characteristics in 3D body data and designate them as keypoints. Thus, a system that converts 3D images into 2D images by creating depth maps was developed to segment specific body parts more efficiently. This approach was chosen to reduce data traffic and processing time associated with the segmentation of 3D images. In the conversion process, the depth information in the 3D images is represented as varying intensities in grayscale, while areas without depth are set to zero, effectively removing color information.

To generate these depth maps, the screen coordinate system was transformed into a 3D coordinate system using a perspective projection matrix [32]. This matrix is used to convert the 3D space, defined by X, Y, and Z coordinates, into a 2D plane comprising only X and Y coordinates. By performing this, the Z coordinate, which represents depth in 3D space, is translated into distances on the 2D plane, allowing us to represent 3D objects in two dimensions [33].

The perspective projection matrix applies a perspective transformation, which means that the same object will appear smaller when it is farther away and larger when it is closer. To express a 3D object in 2D, this conversion process involves a projection plane, a single point through which the transformation is made. During this process, we use ‘aspect’ to define the camera’s field of view, ‘fovy’ to determine the angle of this field, and ‘f’ and ‘n’ to specify the range of *z* values, which represent the projection range. A system was developed to convert 3D object coordinates into 2D for effective body part segmentation. This conversion utilizes the camera’s field of view, specifically the fovy value, which determines the viewing angle. For each angle, we recalculated the x, y, and z coordinates to create a 2D projection through the Projection Plane [34].

The transformation process involves modifying the z-coordinate to align with the x-axis and using trigonometric functions, such as tangent, to derive new coordinate values based on the camera’s visible range and viewing angle. This approach ensures accurate representation of 3D objects in 2D space.

$$tan\left(\frac{fovy}{2}\right)=\frac{1}{-z}$$

$$z=-cot\left(\frac{fovy}{2}\right)$$

Once the coordinates are recalculated, they are arranged into a matrix form. In this matrix, the first column represents the x value, the second the y value, and the third the *z* value. These values are then used to create a depth map by converting each point to grayscale, which is applied uniformly across all pixels.

Based on this depth map, we identified key ‘Seed Points’ corresponding to specific body parts. These points include the end of the head, neck, shoulders, armpits, fingertips, toes, and crotch. The process of identifying these points involves analyzing the body’s topography: the top of the neck marks the end of the head, the thinnest part within the top 30% of the body is identified as the neck, and the shoulder is determined by the inflection point in the curvature from the neck. The armpit is located where a gap appears beneath the shoulder line, and fingertips are detected through a range search in left and right positions. The toes and the crotch end are identified by excluding arms and hands, using a straight-line projection from the arm’s end to the feet, with the transition from the toe tip upwards marking the crotch end.

**Figure 5:**
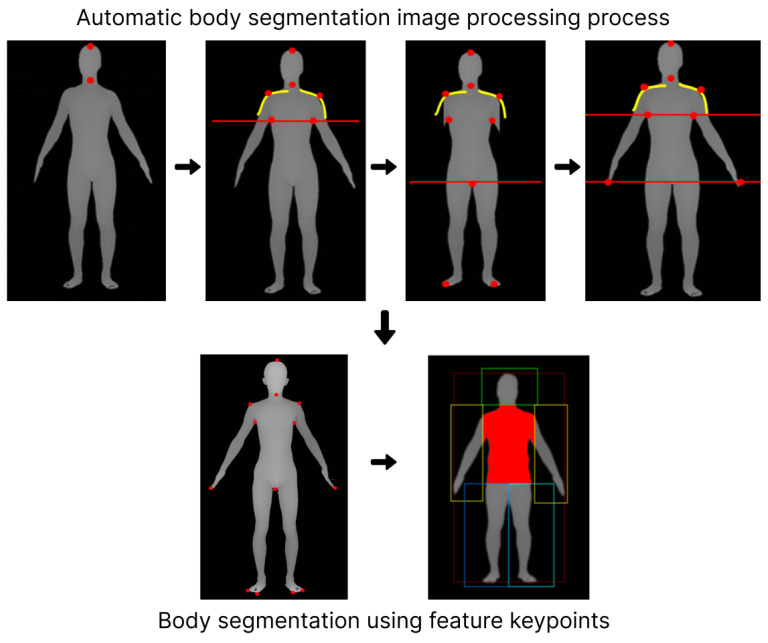
Body feature keypoints detection and segmentation process.

In summary, the system employs a matrix-based approach to transform 3D coordinates into a 2D depth map. From this map, 11 seed points are derived to segment the architecture of the body into distinct parts for analysis, as shown in Figure 5.

#### 4.2.2. 3D Human Body Measurement System

Development of a 3D human body detecting system: A system that divides the body into sections using the keypoint values of the detected body features and derives the values of body circumference, length, and volume using the distances of points for each major body part and provides them to the user was built. Anthropometric survey data from US Army soldiers were utilized, which became the definition of the body standards provided by Size Korea: Summary Statistics Interim Report Body Measurement Standards. In this survey, the body measurements of 1774 men and 2208 women were examined to define the dimensions for military uniforms of the US Army, and the points and definitions of body values for specific body types were clearly presented [35]. To measure body values, utilize the Phong shading model to analyze 3D human data [36]. This model integrates three elements: ambient light, diffuse reflection, and specular reflection. Ambient light refers to indirect light, which also affects surfaces that receive no light. This is expressed as the ambient coefficient and represents the proportion of light that is reflected from a surface. Diffuse reflection represents light spreading in all directions regardless of the direction of light, while specular reflection represents the shiny part of an object or highlight. This is an important factor in making objects look more realistic [37].

In the Phong model, the normal vector of a vertex is calculated as the average of the normal vectors of adjacent faces. This normal vector is used to interpolate the normal vector corresponding to each pixel of the triangle [38]. In the modeling stage, the curved surface is approximated as a plane, but in the rendering stage, the direction of the original curved surface is restored, and the lighting model is calculated. Phong Shading creates a shaded model by globally interpolating normal vectors in various directions for a flat model.

**Figure 6:**
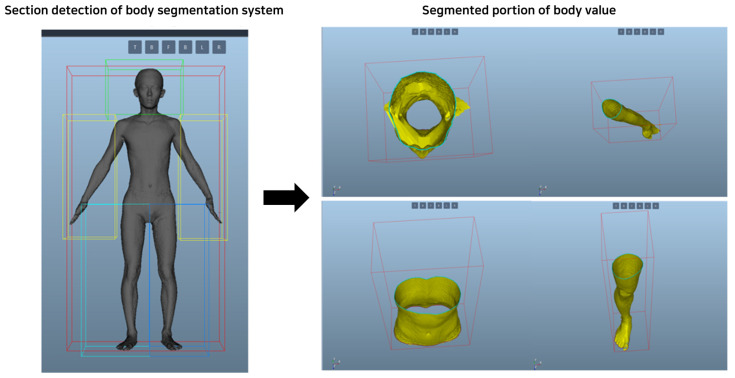
Body segmentation and body value derivation system.

Based on the processed and generated model, the length, circumference, and volume of body parts are measured based on 3D data, as shown in Figure 6. Derive values for the upper and lower perimeters of these data and perform a projection onto the object between these two sections to calculate the total value of the closed section. Using the Phong shading model in this way allows for a more accurate and realistic analysis of 3D human data.

## 5. Results

### 5.1. Select and Create Training Model and Verification Data Adoption

For model comparison, a total of five 3D body generation models, including the proposed model, were used to identify the model that yielded the smallest error while comparing the subjects’ body information with the measured value. For model comparison, a total of five 3D body generation models, including the proposed model, were used to compare the subject’s body information and measurements to identify the model with the smallest error. To determine the generation accuracy of the five models, the body was generated with a 3D scanner, and the body information derived from the machine was used as a reference value and the body values generated by each of the five models were measured using the body segmentation and body value derivation system to compare the accuracy of the five generation models. A cohort of subjects was recruited for model comparison and validation. Data collected about the subject group were used for research through a pledge not to leak data to the outside, and information and 3D body data derived using 2D images and 3D scans were collected. Experimental data for verification were approved by the institutional review board of Dongguk University (DUIRB-202004-11). All methods were carried out in accordance with relevant guidelines and regulations. Informed consent was obtained from all subjects and/or legal guardians that their information/images would be included in an online open-access publication and study. Moreover, we confirmed that informed consent was obtained from all the subjects (for participation).

These models included the SMPL model using a single image, multi-level pixel-aligned impact function for high-resolution 3D human digitization, and 3D current reconstruction neural network, which is a 3D generation model using multiple images. Therefore, we planned to generate 3D body data using each model by setting standards for shooting and height of the 2D input images to find the distance criteria that minimize the errors. These 3D body data generated based on the body values obtained from the 3D scanner are shown in Table 2; the height of the camera and ground were set to 0.5–1.5 m, and the distance between the camera and the measurer was increased from 0.5 m to 4 m. Under these conditions, the PointGAN and 3D-R2N2 models could not derive the body circumference values because of the non-uniform generation of body points in the PointGAN model, as shown in Figure 4. Moreover, the 3D-R2N2 model was evaluated using the values on the X, Y, and Z axes, with a rapidly increasing rate of training as the dimensions increased.

Thus, if the model learns more than 64 × 64 × 64 data points, then the training time and the amount of memory processed increase by an order of 10 compared with the other models, and there is a limit to expressing a body composed of multiple curves. Therefore, the PointGAN and 3D-R2N2 models were excluded from the measurement of the ratio in Table 2 because there is a limit to deriving body values. Accordingly, in this study, SMPL- and PIFuHD-based 3D body generation was performed to set a criterion for minimizing the errors for each photographed distance. After the 3D generation, error verification was performed by determining the ratio of the generated value to the measured value. When 3D data were generated using the SMPL and PIFuHD models as input values, the distance of the camera that minimizes body distortion was used as final input data. In this case, A represents an error in the ratio between the chest and waist circumferences, B represents an error in the ratio of the arm and thigh circumferences, and the distance that minimizes the error of each A and B was selected as the distance of the camera to the final input value. As a result, it was found that the smallest error occurred at the height of 1 m of the camera from the ground and 3 m of the camera from the measurer, and based on this distance, 2D input data were generated and applied to each model. Therefore, SMPL and PIFuHD were utilized in our proposed model to generate 3D body data. These models were selected based on the criteria outlined in Table 2. To assess the accuracy of the generated 3D data, we compared the generation errors with those obtained from a 3D scanner. Additionally, the validation process involved analyzing the ratios of chest and waist circumferences in the 3D models. However, the SMPL model was excluded from further verification because of its significant deviations in body shape values from the expected norms. Consequently, our experiments primarily focused on comparing the PIFuHD model and the model proposed in our study, evaluating their effectiveness in accurately generating 3D body representations.

### 5.2. Model Comparison and Verification

In Section 5.1, the model with the highest generation accuracy among the five models was selected as the final model. Therefore, we chose a multiple high-resolution feature combination generation network. To evaluate this model, we collected real-world data from a total of 214 people (men: 103, women: 111), men and women in their 20s and 30s, for validation. The validation process involved capturing 2D body images according to the standards established in Section 5.1. These 2D images were then input into the 3D body creation model to generate a 3D representation, and information from generated 3D data was extracted using the system built in Section 4.2. The accuracy of the extracted information was verified using a 3D body scanner. 

The body scanner in question is a PFS-304 Series model, and it consists of three sets of camera modules, with the camera at the bottom referred to as number 1, the middle one as number 2, and the camera at the top as camera number 3. Cameras 1 to 3 are correlated with each other. Each camera applies 3D modeling principles that scan the human body in real-time while rotating 360 degrees. Additionally, it provides information on body height, circumference, area, and volume (83 measurement items) by utilizing point clouds of these generated 3D body data. This 3D body scanner is highly accurate, with an error range of less than 1 cm. Model generation errors were identified by comparing the 3D model generated from the 2D image with the 3D model obtained from the scanner. This comparison allowed us to evaluate the accuracy of the network for generating multiple high-resolution feature combinations in generating 3D body models. (https://en.pmt3d.co.kr/PFS-304, accessed on 21 October 2023).

Based on the described methodology, we generated validation data to conduct a comprehensive comparison. This involved analyzing the derived values from both 3D body data created using real-world 2D images and validation data obtained through the body scanner. This comparative analysis enabled us to effectively evaluate the discrepancies and accuracies in the 3D models generated by the network, providing a robust validation of the model’s performance in accurately replicating human body dimensions from 2D images.

Table 3 shows the results of comparing the error values of height, chest circumference, chest volume, waist circumference, and hip circumference between 3D body data generated based on verification data and 3D body data captured with a body scanner. Table 3 presents one value as an example of a comparison of information derived from a total of 111 verification data. Table 3 defines the body values generated and derived from the body scanner as actual values compared with the remaining two models and judged accuracy through errors. The accuracy shown in Table 3 is a value derived from the error of all 111 verification data. Three-dimensional body data were generated for PIFuHD and the model proposed in this study for 111 subjects, and the absolute value (actual value-generated value) was calculated for the derived value for each part, and the average of the error rate was calculated. There may be cases where the generated information can produce better results for certain body types, but we did not consider each of them and calculated the average error rate based on the generated results of each of the 111 groups. Therefore, it is shown in Table 3 as Generate error (%).

## 6. Conclusions

In this study, a 2D input value was applied to 3D generation models to generate body data based on data generated by a 3D scanner, and a generation model that exhibited a body value similar to that obtained using the scanner was developed. Total five models, viz. PointGAN, 3D-R2N2, SMPL, PIFuHD, and the model proposed by us, were used to generate 3D body data using a small number of images. It was observed that it was not possible to derive 3D models for measuring body values using the PointGAN and 3D-R2N2 models, which require many dimensions for calculations. Therefore, the errors in the SMPL, PIFuHD, and proposed model were compared to determine the accuracy of generation. However, the SMPL model generated data with completely different body shape conditions when generating 3D body data, so it was excluded from model verification.

As a result, the average accuracy of measured values and errors of the model proposed in this study was 93%, showing higher generation accuracy than other models. Therefore, it is possible to generate 3D body data using the proposed model, with two front and side 2D images taken with a mobile phone without any 3D equipment. Unlike previously reported 3D-generated models, this is a study that can overcome the limitations of deriving accurate body values using contact sensors or expensive equipment. Our proposed model could generate 3D body data with sizes similar to the actual sizes and resize generated 3D data to measure obesity information based on the body length, circumference, and volume values. Thus, using these 3D data that reflect various body values, volume, and circumferences, obesity information can be derived more accurately compared with the existing obesity indices, such as BMI and WHtR, which are measured using the height, weight, and waist circumferences, and can be used in research to compensate for inaccuracies in body value derivation.

In addition, since the generation model developed in this study can be used to derive 3D body data without time and space constraints, it can be used in healthcare areas that require continuous management, such as obesity and body-shape management. Therefore, in healthcare that requires continuous management, it is possible to determine the state of the body visually and numerically by using only the smartphone camera in a non-contact manner without using complex and expensive equipment or inconvenient sensors. However, the proposed generation model enables the development of one 3D body model through the synthesis of a resized 3D model (such as actual height); thus, all the body parts cannot have similar values. Therefore, 3D body data can be developed by using body proportion elements, although there are limitations in utilizing accurate body length, circumference, and volume values.

Therefore, in the future, it will be necessary to increase the accuracy of 3D generation, capture 2D body images anytime, anywhere, and generate 3D body data to determine obesity and body balance through body length, circumference, and volume.

## Figures and Tables

**Table 1:** Configuration of 3D body modeling data and the average storage requirement per dataset.

Format	3D Geometry	Colors	Textures	Storage
STL	O	X	X	15 MB
OBJ	O	O	O	200 MB
PLY	O	O	X	100 MB
SKP	O	O	O	200 MB
3DS	O	O	O	200 MB

**Table 2:** Comparison of generated data errors through the distance between the camera and the subject.

Height	Distance	SMPL (A, B)	PIFuHD (A, B)
(0.5 m/1 m/1.5 m)	1.5 m	(0.223, 0.326)	(0.174, 0.21)
		(0.251, 0.284)	(0.186, 0.24)
		(0.204, 0.274)	(0.155, 0.232)
(0.5 m/1 m/1.5 m)	2 m	(0.25, 0.124)	(0.142, 0.129)
		(0.197, 0.14)	(0.127, 0.146)
		(0.257, 0.29)	(0.115, 0.178)
(0.5 m/1 m/1.5 m)	2.5 m	(0.114, 0.157)	(0.07, 0.184)
		(0.188, 0.237)	(0.034, 0.24)
		(0.192, 0.16)	(0.094, 0.153)
(0.5 m/1 m/1.5 m)	3 m	(0.081, 0.074)	(0.058, 0.074)
		(0.063, 0.057)	(0.021, 0.034)
		(0.115, 0.047)	(0.045, 0.041)
(0.5 m/1 m/1.5 m)	3.5 m	(0.125, 0.155)	(0.065, 0.081)
		(0.245, 0.156)	(0.242, 0.213)
		(0.162, 0.122)	(0.172, 0.084)
(0.5 m/1 m/1.5 m)	4 m	(0.224, 0.172)	Data broken
		(0.12, 0.149)	
		(0.092, 0.157)	

**Table 3:** Comparison of body-derived values between 3D scanner and generated models.

Measurement Group	Actual Values	PIFuHD	Multi-High-Resolution Feature Combination Generation Network	Generation Error (%) (PIFUHD/Multi-High-Resolution Feature Combination Generation Network)
Height	162.4 cm	-	-	-
Chest circumference	111.02	95.4 cm	107 cm	15.7%/7.5%
Chest volume	12 L	9.3 L	11.2 L	20.5%/6.2%
Waist circumference	104.08 cm	91.1 cm	100.2 cm	25.9%/8.2%
Hip circumference	101.86	90.4 cm	96.5 cm	30.2%/5.4%
Accuracy	100%	85%	93%	

## Data Availability

The data presented in this study are available on request from the corresponding author.
